# Fine-Grained Spatiotemporal Analysis of the Impact of Restricting Factories, Motor Vehicles, and Fireworks on Air Pollution

**DOI:** 10.3390/ijerph17134828

**Published:** 2020-07-04

**Authors:** Mei Yang, Hong Fan, Kang Zhao

**Affiliations:** 1 State Key Laboratory of Information Engineering in Surveying, Mapping and Remote Sensing, Wuhan University, Wuhan 430079, China; amyyang202@163.com (M.Y.); kzhao@whu.edu.cn (K.Z.); 2 Collaborative Innovation Center of Geospatial Technology, Wuhan University, Wuhan 430079, China

**Keywords:** particulate matter, public health, air quality, random forest, COVID-19

## Abstract

Aiming at improving the air quality and protecting public health, policies such as restricting factories, motor vehicles, and fireworks have been widely implemented. However, fine-grained spatiotemporal analysis of these policies’ effectiveness is lacking. This paper collected the hourly meteorological and PM_2.5_ data for three typical emission scenarios in Hubei, Beijing–Tianjin–Hebei (BTH), and Yangtze River Delta (YRD). Then, this study simulated the PM_2.5_ concentration under the same meteorological conditions and different emission scenarios based on a reliable hourly spatiotemporal random forest model ($R^{2}$ exceeded 0.84). Finally, we investigated the fine-grained spatiotemporal impact of restricting factories, vehicles, and fireworks on PM_2.5_ concentrations from the perspective of hours, days, regions, and land uses, excluding meteorological interference. On average, restricting factories and vehicles reduced the PM_2.5_ concentration at 02:00, 08:00, 14:00, and 20:00 by 18.57, 16.22, 25.00, and 19.07 $\mu g/m^{3}$, respectively. Spatially, it had the highest and quickest impact on Hubei, with a 27.05 $\mu g/m^{3}$ decrease of PM_2.5_ concentration and 17 day lag to begin to show significant decline. This was followed by YRD, which experienced a 23.52 $\mu g/m^{3}$ decrease on average and a 23 day lag. BTH was the least susceptible; the PM_2.5_ concentration decreased by only 8.2 $\mu g/m^{3}$. In addition, influenced by intensive human activities, the cultivated, urban, and rural lands experienced a larger decrease in PM_2.5_ concentration. These empirical results revealed that restricting factories, vehicles, and fireworks is effective in alleviating air pollution and the effect showed significant spatiotemporal heterogeneity. The policymakers should further investigate influential factors of hourly PM_2.5_ concentrations, combining with local geographical and social environment, and implement more effective and targeted policies to improve local air quality, especially for BTH and the air quality at morning and night.

## 1. Introduction

Air pollution is a great challenge to human health worldwide. The atmospheric particulate matter, especially particulate matter with a diameter of less than 2.5 μm (PM_2.5_), can be inhaled into the human body due to its tiny diameter. Therefore, it may cause respiratory and cardiovascular diseases [1,2,3]. Besides the physical damage, epidemiological and toxicological studies report that PM_2.5_ pollution is associated with depression and anxiety symptoms [4,5] and the oxidative stress and inflammation induced by PM_2.5_ may cause brain injury [6,7]. Therefore, it is urgent for governments to implement a series of policies to control the air pollution level effectively and to protect public health. Among them, restricting factories, motor vehicles, and fireworks are some of the most common measures in China and full recognition of their effectiveness is important.

Many studies have investigated the correlation between factories, motor vehicles, and fireworks with PM_2.5_ concentration. Based on their research method, they can be divided into two categories—namely, the multivariate receptor and models and econometric analysis models [8]. The multivariate receptor models analyze the components of PM_2.5_ from the perspective of chemistry and physics to identify its source. For instance, Wang et al. collected meteorological data and compositional data of PM_2.5_ in a monitoring station located in Shanghai over a month [9]. The emission sources of PM_2.5_ were identified using the positive matrix factorization method, including secondary nitrate, secondary sulfate, vehicular/industrial emissions, and coal combustion. Yu et al. recorded the hourly concentrations of 18 elements of PM_2.5_ at an urban site of Nanjing during 2017 [10]. They found that some specific element’s concentrations would increase when the traffic activities, fireworks, and sandstorm events occurred. Similar studies were conducted in Beijing, Guangzhou, and other major cities in China [11,12,13,14,15]. These studies demonstrated that air pollution in China is one of the highest in the world. The possible sources consist of coal combustion, soil dust, traffic emissions, secondary inorganic aerosols, and emissions from industrial processes and wood combustion. Feng et al. and Wang et al. conducted PM_2.5_ concentration monitoring experiments during the Spring Festival in Xinxiang and Xiamen, respectively [16,17]. The chemical analysis results indicated that the burning of fireworks during the festival worsens air quality in a short period. This was also confirmed in the United States. Seidel et al. collected the systematic observations of 315 monitoring stations across the United States over multiple years and the results showed that during the Independence Day holiday, the hourly PM_2.5_ concentration of one site adjacent to fireworks climbed to ~500 $\mu g/m^{3}$ [18]. Although the multivariate receptor methods contribute much to the identification of the correlation between factories, vehicles, and fireworks with PM_2.5_, most of these studies were conducted in a local region with very limited monitoring equipment. Thus, the conclusions drawn from these investigations do not provide a basis for implementing policies in a large area.

The econometric models use many economical methods to explore the impact of factories and vehicles on PM_2.5_ concentration. Xu et al. and Huang et al. collected statistical panel data of China over 10 years and used the stochastic impacts by regression on population, affluence, and technology (STIRPAT) model to explore the driving factors of PM_2.5_ [19,20]. Cheng et al. and Luo et al. improved the spatial scale to the civic level and obtained many details of the driving forces [8,21]. Aside from the STIRPAT model, other economic methods were also adopted to explore the driving forces. Ma et al. analyzed the driving factors of fog and haze in 152 cities in China using the spatial autoregressive model [22]. Wu et al. collected data from 74 cities with PM_2.5_ monitoring stations in 2013 and 2014 and explored the determinants of PM_2.5_ using the random effect model [23]. These studies demonstrated that on an economic development level, urbanization level, coal consumption, motor vehicles, and population size are key influencing factors of PM_2.5_. Wang et al. used a spatial panel Dubin model to investigate the relationship between PM_2.5_ and six socioeconomic factors during 2015~2017 in Beijing–Tianjin–Hebei (BTH) [24]. It was found that the urbanization rate has a negative effect on PM_2.5_, which may be due to the stricter environmental regulations than before. All of these studies demonstrated the urgency and importance in implementing emission control measures and evaluating their effectiveness. Although Zhang et al. and Ding et al. have confirmed the impact of the active clean air policies during 2013–2017 on reducing PM_2.5_, the spatiotemporal resolution in these studies were low, meaning the fine-grained spatiotemporal impact was not investigated [25,26]. However, with different land uses, the PM_2.5_ pollution level and socioeconomic condition varied; such variation leads to differences in the impact of human factors with changing land-use trends [27]. Since PM_2.5_ concentration varies over the course of the whole day, the effect of restricting industries and traffic may also vary with different hours. In addition, whether the restriction of factories and vehicles can take effect on improving air quality immediately or whether this requires some time to show a significant effect is also valuable to investigate. Since most of the economic studies are conducted based on provincial or civic data, the abovementioned fine-grained spatiotemporal effect has not been fully explored.

The variations in meteorological conditions can dominate the monthly PM_2.5_ variations on a regional scale [25]. To fully recognize the spatiotemporal impact of the restriction of factories and motor vehicles on PM_2.5_, the meteorological interference must be eliminated. Therefore, studies on the modeled PM_2.5_ pollution level under the same meteorological conditions but different emission conditions, such as open or total closure of factories and commuting, are required. First, the scenario of closing factories and vehicles, which is ordinarily impossible to realize, needs to be created. The opportunity to do this presented itself in late December 2019, when an outbreak of a novel coronavirus (COVID-19) emerged in Wuhan, China and rapidly spread across China [28]. Considering its strong infectious ability, Chinese provinces and regions carried out a level 1 response to major public health emergencies, which required people to stop attending school and going to work. Moreover, the factories were closed [28,29,30,31,32,33,34]. These extreme response measures provided the emission scenario of factories being shut down and a reduction in travel. The remaining problem is how to retrieve the PM_2.5_ concentration under the same meteorological condition but different emission scenarios. The widely used retrieval models include chemistry transport models (CTM), air pollution dispersion models, and statistical models. CTM retrieve PM_2.5_ based on the transport mechanism and physical as well as chemistry reactions of air pollutants [35,36]. Dispersion models, such as the Gaussian dispersion model and Lagrangian dispersion model, simulate the surface air quality according to the estimation of the impact of the point, line, volume, and area emission sources [37,38]. For the purpose of comparing the impact of different emission scenarios on air pollution, both CTM and dispersion models require the corresponding meteorological data and concrete emission inventory of different time periods. However, due to the complex composition of emission sources, rapid technological update, and difficulty in obtaining relevant information, accurate and up-to-date emission inventory is difficult to collect. The most widely used emission inventory for China is the Multi-resolution Emission Inventory of China (MEIC, http://www.meicmodel.org/) and its newest version is 2015. Therefore, it is difficult to obtain the emission inventory under different time periods, meaning the CTM and dispersion models cannot be applied for retrieving air pollution under different emission scenarios. Therefore, this paper adopted the random forest (RF) model, which is a widely used statistical model in PM_2.5_ retrieval studies with high accuracy, to simulate the PM_2.5_ concentration under the same meteorological conditions but different emission scenarios [39].

The aim of this paper is to investigate the fine-grained spatiotemporal effect of the restrictions of factories, vehicles, and fireworks on air pollution. Hubei province, which is the worst hit province by COVID-19, together with the heavily contaminated region, Beijing–Tianjin–Hebei (BTH), and the Yangtze River Delta (YRD) region were chosen as the study areas. The PM_2.5_ concentration and meteorological data for three time periods were collected as follows: the COVID-19 isolation period (time period 1: 21 January 2020–20 February 2020), the previous month before the isolation (time period 2: 21 December 2019–20 January 2020), and the same period in the lunar calendar of last year (time period 3: 1 February 2019–3 March 2020). The three time periods represent three typical emission scenarios, namely, shutting down factories and vehicles (SD), normal commute (NC), and normal spring festival (NSF). The severe air pollution is not only a natural phenomenon, but also a man-made contamination caused by long-term unhealthy economic activities [22,40,41]. Thus, the influence of weather must be eliminated when studying the impact of emission control measures. This paper used the meteorological data and PM_2.5_ concentration data during NSF and NC periods to develop two separate spatiotemporal random forest (STRF) models to simulate the correlation between meteorological factors and PM_2.5_ concentration under the specific emission scenarios of the corresponding periods. Then, the meteorological data of the SD period was inputted in two STRF models to obtain the simulated PM_2.5_ concentration under the weather condition of SD together with emission scenarios of NSF and NC. By comparing the measured PM_2.5_ concentrations during SD with the simulated PM_2.5_ concentration under NSF and NC emission scenarios, we determined the pollution difference brought by the change of emission scenarios and the impact of emission control measures, including shutting down factories and vehicles. Significant spatiotemporal heterogeneity of the impact of factories, vehicles, and fireworks was characterized according to the results.

The main objectives and contributions can be summarized as follows: (a) Develop reliable retrieval models to simulate the PM_2.5_ level under the same meteorological conditions and different emission scenarios. The simulated PM_2.5_ shared the same meteorological background, thus the comparison of them can eliminate the meteorological interference and therefore, it is more accurate in evaluating the effect of different emission conditions on PM_2.5_ than directly comparing the PM_2.5_ observed under the three emission scenes. (b) Compare the difference of the PM_2.5_ variations that were induced by the restriction of factories, vehicles, and firework from the perspective of different hours and days. The temporal resolution is finer than month and year, so the results can fill in the deficiencies of previous large-scale studies that were conducted based on panel data. (c) Spatially, compare the difference of the PM_2.5_ concentration variations in different regions and land uses. The influence of fireworks on PM_2.5_ was studied in an extensive spatial range, breaking through the limitations of previous studies, which relied on several limited stations.

## 2. Materials and Methods

### 2.1. Development of Emission Scenarios

Hubei province is in the middle area of China and is one of the most important transportation junctions of China due to its location. In late December of 2019, COVID-19 broke out in Wuhan, Hubei. On the night of 20 January 2020, Academician Zhong Nanshan, who is the head of the high-level expert group appointed by China’s National Health Commission to fight COVID-19, announced in an interview that the phenomenon of human-to-human transmission of COVID-19 had been confirmed. This announcement signaled the start of the public’s awareness of the contagious nature of COVID-19. People began their self-quarantine at home to avoid coming into contact with others. Considering that the disease can be transmitted by direct contact and droplets, two days after the announcement, Chinese provinces and regions launched a level 1 response to major public health emergencies [28,29,30,32]. Under this policy, people were compulsorily required to stay at home to the greatest extent. The factories and schools were closed and the traffic flow was reduced greatly [33,34]. Such a situation lasted until 23 February 2020 [31]. This emergency transformed China into a natural laboratory that can be used to study the impact of the restriction of factories and traffic on the alleviation of air pollution.

To compare the air pollution level under different emission scenarios, we selected three typical periods, as follows: the COVID-19 isolation period (time period 1: 21 January 2020–20 February 2020), the previous month before the isolation (time period 2: 21 December 2019–20 January 2020), and the same period in the lunar calendar of last year (time period 3: 1 February 2019–3 March 2020). These three study periods represented three emission scenarios and are described in Table 1.

Time period 1 represents the emission scenario of SD. During this period, owing to subjective wishes and objective government policies, people tried their best to stay at home, resulting in the near complete shutdown of traffic and factories. In addition, this period was in winter, thus the winter heating was functioning as normal. The 2019 Chinese Spring Festival was held on 25 January 2020, and during this event, fireworks were present. Time period 2 represented the emission scenario of NC, in which the factories and vehicles were open and operational as normal. This period was in winter and the Spring Festival was not held. Thus, the heating was normal and firework displays did not occur. Time period 3 represents the emission scenario of the NSF. During this period, emission from fireworks existed. The heating, factories, and vehicles were nearly normal, regardless of the short holiday of the Spring Festival. By comparing air pollution under SD and NSF emission scenarios, the effect of shutting down factories and vehicles was determined. By further comparing air pollution under SD and NC, the effect of fireworks was determined.

### 2.2. Data

BTH refers to the region comprising Beijing, Tianjin, and Hubei provinces. YRD refers to the region containing Shanghai, Zhejiang, Jiangsu, and Anhui provinces. BTH and YRD are both strategically and economically important regions in China, with a large number of citizens. According to China Statistical Yearbook, BTH and YRD have 112.7 million and 225.36 million people occupying 8.08% and 16.15% of the national population, respectively [42]. Influenced by the rapid urbanization and unhealthy ways of economic development, air quality in BTH and YRD has declined in the past years, leading to serious public exposure to severe air pollution.

**Figure 1:**
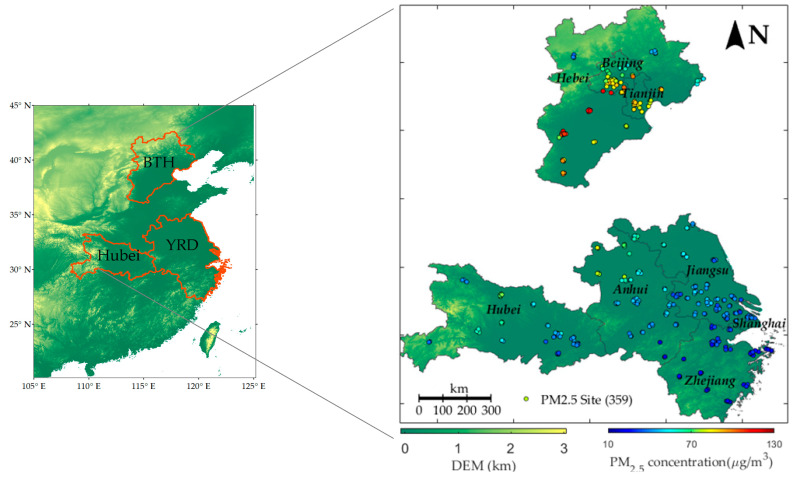
Study areas and spatial distribution of mean PM_2.5_ concentration.

In this paper, the study areas were BTH, YRD, and Hubei province. Hubei province is the area worst hit by COVID-19. We collected PM_2.5_ data of the study area in three periods listed in Table 1 from the China National Environmental Monitoring Center (http://106.37.208.233:20035/) and Beijing Municipal Environmental Monitoring Center (http://www.bjmemc.com.cn/). The stations owning over 20% missing values during the months of the study periods were omitted. In total, 359 out of 422 stations were retained and the missing data percentage of the remaining stations was 3.49%. The remaining missing observations were interpolated via linear interpolation based on the PM_2.5_ observations of the previous and later hours if both of them existed. Otherwise, interpolation was conducted via the inverse distance squared method according to the observations of other stations. The accuracy of the interpolation procedure was validated via 10-fold cross validation and the average mean absolute error was 4.78 $\mu g/m^{3}$. Figure 1 shows the study area’s locations and spatial distribution of mean PM_2.5_ concentration during SD. From Figure 1, PM_2.5_ showed a worsening pollution trend from south to north, except for Northern Hebei.

The meteorological data of the study area during the study periods were collected from the global Copernicus Atmospheric Monitoring Services (CAMS) production system of the European Centre for Medium-Range Weather Forecasts (ECMWF) (http://apps.ecmwf.int/datasets/data/cams-nrealtime/levtype=sfc/). This system combines the satellite observations and uses the Integrated Forecasting System (IFS) to model the meteorological fields and the processes of atmosphere composition. The reliability of the CAMS products and IFS is evaluated through a series of Comprehensive Evaluation and Quality Assurance reports and some regional analysis [43,44]. The CAMS near-real-time dataset can provide the meteorological data with a spatial resolution of 0.125°, which is higher than that of the meteorological observations from limited monitoring stations. Therefore, we collected the meteorological data from the CAMS dataset, including the U component of wind, V component of wind, mean sea level pressure, dew point temperature, and temperature; all of these were key meteorological factors of PM_2.5_ [45,46]. ECMWF provided meteorological data at 02:00, 08:00, 14:00, and 20:00 (Beijing Time, BJT). Thus, only the PM_2.5_ data observed at these hours were considered. The meteorological data were resampled to about 5 km spatial resolution and the PM_2.5_ monitoring stations were matched with its nearest grid. The average PM_2.5_ observations were calculated for each grid, which contains PM_2.5_ monitoring sites, and they were combined with the meteorological data of the grids and observation time to form the whole study samples. All data were centralized and standardized in accordance with the following equation before the matching procedure:

(1) $$x^{*}=\frac{x-\bar{x}}{\sigma },$$

where x and $x^{*}$ represent the original and transformed observation of a variable, respectively. $\bar{x}$ and σ are the mean and standard deviation of all observations, respectively.

### 2.3. PM_2.5_ Modeling and Evaluation Methods

This paper aimed to identify the spatiotemporal effect of restricting factories and vehicles during SD on the alleviation of air pollution. PM_2.5_ pollution is not only a natural phenomenon influenced by meteorological conditions, but is also influenced by severe anthropogenic emissions [8,22,40,41,47,48]. Thus, the relationship between PM_2.5_ meteorological variables is not constant but is changing over time [24]. Therefore, we assumed that the developed regression model trained based on the PM_2.5_ and meteorological data collected from a specific period represents the relationship between them under the emission scenario of the specific period. Consequently, inputting the same meteorological dataset into different regression models that were developed based on different datasets, the results represent the expected PM_2.5_ level under the same meteorological conditions and different emission scenarios of different datasets. Therefore, the comparisons of the expected PM_2.5_ concentration can eliminate the meteorological interference and directly illustrate the impact of different emission scenarios on PM_2.5_.

**Figure 2:**
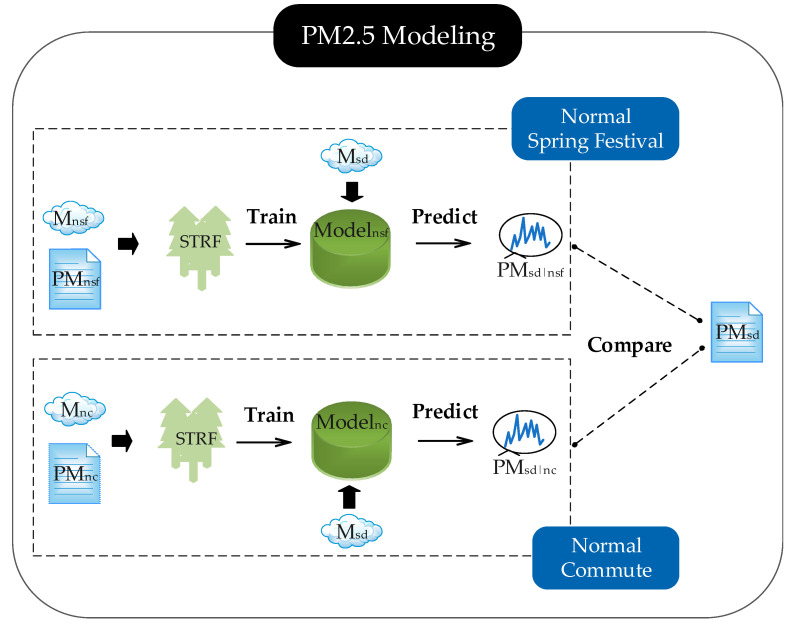
Workflow of PM_2.5_ modeling.

To achieve this goal, this paper established a PM_2.5_ modeling framework to compare the PM_2.5_ concentrations under different emission scenarios. The workflow of PM_2.5_ modeling is illustrated in Figure 2. $M_{sd}$, $M_{nc}$, and $M_{nsf}$ represent the meteorological data of SD, NC, and NSF periods, respectively, and $PM_{sd}$, $PM_{nc}$, and $PM_{nsf}$ represent the PM_2.5_ observations of the corresponding period. The “STRF” model refers to the developed spatiotemporal random forest model in this paper, which will be explained in detail later.

Step1. The meteorological data and PM_2.5_ observations of the NSF period ($M_{nsf}$ and $PM_{nsf}$) and NC period ($M_{nc}$ and $PM_{nc}$) were inputted into the STRF models, respectively.
Step2. The STRF models were trained in R software using the “randomForest” function. Then, the trained model $Model_{nsf}$ and $Model_{nc}$ were generated with the following equations,
  (2) $$Model_{nsf}:\;PM_{nsf}=f_{nsf}\left(M_{nsf}\right),$$
  (3) $$Model_{nc}:\;PM_{nc}=f_{nc}\left(M_{nc}\right),$$
  where $f_{nsf}$ and $f_{nc}$ represent the nonlinear relationship between meteorological data and PM_2.5_ concentration under the specific emission scenario of NSF and NC, respectively. The training process of STRF is introduced later.
Step3. Then, the meteorological data of SD ($M_{sd}$) was inputted into $Model_{nsf}$ and $Model_{nc}$ to generate the predictions of PM_2.5_ concentration using the following equations.
  (4) $${\hat{PM}}_{sd|nsf}=f_{nsf}\left(M_{sd}\right),$$
  (5) $${\hat{PM}}_{sd|nc}=f_{nc}\left(M_{sd}\right)$$
  ${\hat{PM}}_{sd|nsf}$ and ${\hat{PM}}_{sd|nc}$ represent the expected PM_2.5_ pollution level induced by the weather condition of SD under the emission condition of NSF and NC, respectively.
Step4. By comparing $PM_{sd}$, ${\hat{PM}}_{sd|nsf}$, and ${\hat{PM}}_{sd|nc}$ from multiple perspectives, we analyzed how the restriction of factories and vehicles during SD affected the PM_2.5_ pollution level both spatially and temporally. It is noted that $PM_{sd}$, ${\hat{PM}}_{sd|nsf}$, and ${\hat{PM}}_{sd|nc}$ represent the observed or predicted PM_2.5_ concentrations induced by the meteorological condition of SD under the emission scenarios of SD, NSF, and NC, respectively. The meteorological and emission scenarios of $PM_{sd}$, ${\hat{PM}}_{sd|nsf}$, and ${\hat{PM}}_{sd|nc}$ are listed in Table 2.

The STRF model is a spatiotemporal random forest model developed in this study for simulating the PM_2.5_ concentration. Its core idea is to introduce spatiotemporal heterogeneity of PM_2.5_ into ordinary RF by introducing spatiotemporal factors. To deal with the diurnal variation of PM_2.5_, this paper divided the PM_2.5_ observations and meteorological data into four groups according to the observation time, namely, 02:00, 08:00, 14:00, and 20:00 (BJT). Then, for the data of each group, an STRF model was developed to simulate the correlation between PM_2.5_ with meteorological and location variables at the specific hour. The STRF model is written as follows:

(6) $${PM}_{2.5|hour}=f(U_{wind|hour},V_{wind|hour},{msl}_{hour},d_{2m|hour},t_{2m|hour},lat,lon),$$

where the subscript of “*hour*” represents the observations at 02:00, 08:00, 14:00, or 20:00 (BJT) and f is nonlinear function. The influencing variables of PM_2.5_ included meteorological variables (U component of wind ($U_{wind|hour}$), V component of wind ($V_{wind|hour}$), mean sea level pressure ($msl_{hour}$), dew point temperature ($d_{2m|hour}$) and temperature ($t_{2m|hour}$), and location variables (the latitude and longitude of the center of the study grid)). The spatial heterogeneity of PM_2.5_ was considered in the model. The training process of the STRF model is described as follows.

Step 1. The sample size of the training dataset was assumed to be *n*. Then, the algorithm first drew *n* bootstrap samples from the whole training dataset.
Step 2. These samples were used to grow an unpruned regression tree. At each node, the best split factor was chosen from M randomly selected candidate factors to make the uncertainty of the split subsets reach the least.
Step 3. The abovementioned steps were repeated ntree times to grow ntree trees. Predictions were made by averaging the predictions of ntree trees. Considering that PM_2.5_ concentration must be positive values, the final prediction of PM_2.5_ concentration was determined as the maximum value between the original prediction and 0.

Both $Model_{nsf}$ and $Model_{nc}$ were trained based on the above process and ${\hat{PM}}_{sd|nsf}$ as well as ${\hat{PM}}_{sd|nc}$ were generated through inputting $M_{sd}$ to $Model_{nsf}$ and $Model_{nc}$, respectively.

Three evaluation criteria were used to verify the accuracy of the STRF model in retrieving the PM_2.5_ concentration, including the root mean square error (RMSE), mean absolute error (MAE), and R square ($R^{2}$). RMSE and MAE represent the prediction error of the retrieval model. The less RMSE and MAE are, the higher the accuracy of the retrieval model. $R^{2}$ describes the fitting degree of the STRF model to the PM_2.5_ observations, and a high value indicates increased model reliability. The formulas of these criteria are defined as follows:

(7) $$\mathrm{RMSE}=\sqrt{\frac{1}{n}\sum_{i=1}^{n}{\left(y_{i}-y_{i}^{*}\right)}^{2}},$$

(8) $$\mathrm{MAE}=\frac{1}{n}\sum_{i=1}^{n}\left|y_{i}-y_{i}^{*}\right|,$$

(9) $$R^{2}={\left(\frac{Cov\left(Y,\;Y^{*}\right)}{\sqrt{Var\left(Y\right)}\sqrt{Var\left(Y^{*}\right)}}\right)}^{2}\times 100\%,$$

where *n* is the sample size;$\;y_{i}$ and $y_{i}^{*}$ are the observed and predicted PM_2.5_ concentration, respectively. Cov(·) represents the covariance, Var(·) is the variance, and Y as well as $Y^{*}$ represent the observed sequence and predicted sequence of PM_2.5_ concentration.

## 3. Results

The experimental data in this study included the PM_2.5_ concentration, meteorological data, and location data of stations of BTH, YRD, and Hubei province in the following three time periods: 21 January 2020–20 February 2020 (SD), 21 December 2019–20 January 2020 (NC), and 1 February 2019–3 March 2019 (NSF). The emission situations of the three periods are introduced in Table 1. Limited by ECMWF data, only PM_2.5_ observations at 02:00, 08:00, 14:00, and 20:00 (BJT) were considered. For each hour in the NC and NSF period, a particular STRF model was developed to simulate the correlation between meteorological data and PM_2.5_ for this hour and period. In total, there were eight STRF models developed. The number of trees and randomly selected features in STRF were set as 200 and 2, respectively. According to the emission condition description in Table 1 and the data description in Table 2, the comparison between $PM_{sd}$ and ${\hat{PM}}_{sd|nsf}$ indicated the effect of shutting down factories and vehicles on the alleviation of air pollution; by further comparing $PM_{sd}$ with ${\hat{PM}}_{sd|nc}$, the effects of fireworks in the Spring Festival were inferred.

Section 3.1. introduces the reliability of the STRF model in retrieving the PM_2.5_ concentration, which assured the feasibility of using the predicted results of STRF models to serve as the simulated PM_2.5_ under specific meteorological and emission conditions. Section 3.2 and Section 3.3 analyze the temporal and spatial impact of fireworks, factories, and vehicles on air pollution in BTH, YRD, and Hubei province, respectively. Furthermore, considering that land-use categories affect the emission level, the correlation between different land-use categories with air pollution was investigated.

### 3.1. Reliability of the STRF Model

This subsection introduces the prediction accuracy of eight STRF models. Table 3 shows the average RMSE, MAE, and R^2^ results of the 10-fold cross validation of STRF models built for 02:00, 08:00, 14:00, and 20:00 (BJT) under NC and NSF. In addition, the “Obs.” column in Table 3 also shows the average PM_2.5_ concentration of different regions and hours during the NSF and NC period. From the view of variations within a day, the PM_2.5_ concentrations at 08:00 and 14:00 were lower than that at 02:00 and 20:00, which indicates that the air quality in the daytime was better than at night. The STRF models at 14:00 were the most accurate among the four hour models, with the highest R^2^ of the whole study area reaching 0.88 and 0.89 for NSF and NC, respectively. The performance of the models at three other hours were similar, with the RMSE of the whole study area being around 20 and 19 $\mu g/m^{3}$ and MAE being around 13 and 12 $\mu g/m^{3}$ for NSF and NC, respectively. From the perspective of regions, air pollution was the most severe in BTH, followed by Hubei, and the air quality in YRD was the best. At the same time, the RMSE and MAE were larger in BTH than YRD and Hubei, which is due to the fact that more severe air pollution usually implies more complex emission sources and naturally, the PM_2.5_ concentration was more difficult to make accurate predictions. Notably, for all models under different times and scenarios, the R^2^ of the whole study area exceeded 0.84, indicating that the developed STRF models could explain over 84% of the PM_2.5_ concentration variation. The high prediction accuracy of STRF models constructed a solid foundation for using the retrieval results of STRF models to serve as the simulated air pollution level under the meteorological conditions of SD and emission conditions of NC or NSF.

### 3.2. Temporal Analysis of the Effect of Fireworks, Factories, and Vehicles

Table 4 shows the average difference of PM_2.5_ concentration under the emission scenarios of SD, NC, and NSF. The italicized numbers implied that the gap exceeds the MAE range of the corresponding STRF model for NSF or NC periods. As described in Table 2, $PM_{sd}$, ${\hat{PM}}_{sd|nsf}$, and ${\hat{PM}}_{sd|nc}$ were observed or predicted under the meteorological conditions of SD and different emission scenarios, such as SD, NSF, and NC. The negative values in Table 4 indicate that compared with NSF or NC, the emission control measures during the SD period declined PM_2.5_ concentration to a certain degree. According to the emission scenarios described in Table 1, the main difference in the emission during SD and NSF was whether the factories and vehicles were shut down due to COVID-19. The main difference in the emission during SD and NC was the presence or absence of fireworks and whether the factories and vehicles were shut down.

The results of $PM_{sd}-{\hat{PM}}_{sd|nsf}$ indicated the effect of restricting factories and vehicles during SD on the decline of the PM_2.5_ concentration. The difference of $PM_{sd}-{\hat{PM}}_{sd|nsf}$ and $PM_{sd}-{\hat{PM}}_{sd|nc}$ implied the effect of fireworks at the Spring Festival. Some useful conclusions can be drawn from Table 4. (a) For 02:00, 08:00, 14:00, and 20:00, restricting factories and vehicles during SD caused PM_2.5_ to decrease by 18.57, 16.22, 25.00, and 19.07 $\mu g/m^{3}$, respectively, on average. The effect was the least for BTH and the largest for YRD and Hubei at 14:00. Especially for Hubei province, the restriction of factories and vehicles during SD induced the PM_2.5_ concentration at 14:00 to show a decrease of 36.28 $\mu g/m^{3}$. (b) From the perspective of regions, restricting factories and vehicles decreased the PM_2.5_ concentration by 8.20, 23.52, and 27.05 $\mu g/m^{3}$ for BTH, YRD, and Hubei, respectively, on average. The smallest $\left|{\mathrm{PM}}_{\mathrm{sd}}-{\hat{PM}}_{sd|nsf}\right|$ were found in BTH and all values were within the MAE range. This finding illustrated that in BTH, the closure of factories and vehicles during SD did not significantly affect the improvement of air quality. On the contrary, all of the values of $PM_{sd}-{\hat{PM}}_{sd|nsf}$, in YRD and Hubei, they exceeded the MAE range according to the STRF model, indicating that the effect of shutting down factories and vehicles during SD was significant. (c) By comparing mean $PM_{sd}-{\hat{PM}}_{sd|nc}$ with $PM_{sd}-{\hat{PM}}_{sd|nsf}$, it was found that the set off of fireworks increased PM_2.5_ by 10.94, 6.03, and 11.46 $\mu g/m^{3}$ for BTH, YRD, and Hubei, respectively, on average.

**Figure 3:**
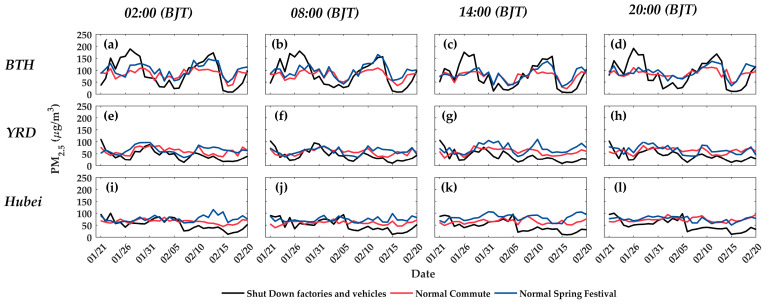
Temporal trend of the observed or predicted PM_2.5_ concentration during the period of the shut down of factories and vehicles under different emission scenarios. The black, red, and blue lines represent the PM_2.5_ concentration under shutting down factories and vehicles (SD), normal commute (NC), and spring festival (NSF), respectively. (**a–l**) represent the results of BTH, YRD, and Hubei at 02:00, 08:00, 14:00, and 20:00 (BJT), respectively.

Figure 3 exhibits the temporal trend of the observed or predicted PM_2.5_ concentration in the SD period under different emission scenarios. The black lines represent the observed PM_2.5_ values ($PM_{sd}$) and the red and blue lines represent the predicted PM_2.5_ values under NC and NSF (denoted by ${\hat{PM}}_{sd|nc}\;\mathrm{and}\;{\hat{PM}}_{sd|nsf}$), respectively. (a) The temporal trend of observed PM_2.5_ concentration varied in different regions. For the BTH region, two peaks occurred around 26 January and 12 February, reaching almost 200 $\mu g/m^{3}$. In contrast, the observed PM_2.5_ concentrations $PM_{sd}$ gradually decreased until less than 50 $\mu g/m^{3}$ for YRD and Hubei, without any large peaks. (b) The difference between $PM_{sd}$ with ${\hat{PM}}_{sd|nc}\;\mathrm{and}\;{\hat{PM}}_{sd|nsf}$ differs in three regions. For BTH, in the days around two peaks, $PM_{sd}$ exceeded ${\hat{PM}}_{sd|nc}\;\mathrm{and}\;{\hat{PM}}_{sd|nsf}$; the two accidental peaks were the main reason for the smaller mean $PM_{sd}-{\hat{PM}}_{sd|nc}$ and $PM_{sd}-{\hat{PM}}_{sd|nsf}$ results for BTH in Table 4. For YRD and Hubei, ${\hat{PM}}_{sd|nc}$ and ${\hat{PM}}_{sd|nsf}$ in YRD and BTH fluctuated at around 70 $\mu g/m^{3}$ on the whole, indicating that under the meteorological condition of the SD period, PM_2.5_ concentrations were expected to be around 70 $\mu g/m^{3}$ under the emission scenarios of NC and NSF. Combined with the decreasing trend of observed PM_2.5_, it could be concluded that restricting factories and vehicles during SD gradually affected the air pollution level and reduced PM_2.5_ concentration. The turning point was the time when ${\mathrm{PM}}_{\mathrm{sd}}$ started to show significantly lower values compared with ${\hat{PM}}_{sd|nc}$ and ${\hat{PM}}_{sd|nsf}$. This was the time when the closure of factories and vehicles started to essentially decrease the air pollution level. The turning points for YRD and Hubei were different. For the YRD region, from 13 February, most $PM_{sd}$ were lower than ${\hat{PM}}_{sd|nc}$ and ${\hat{PM}}_{sd|nsf}$. For the Hubei region, the turning point was on 7 February. This illustrated that there was a delay in shutting down factories and vehicles to have significant effects on alleviating air pollution and the delay time was 23 and 17 days for YRD and Hubei, respectively.

### 3.3. Spatial Analysis of the Effect of Fireworks, Factories, and Vehicles

**Figure 4:**
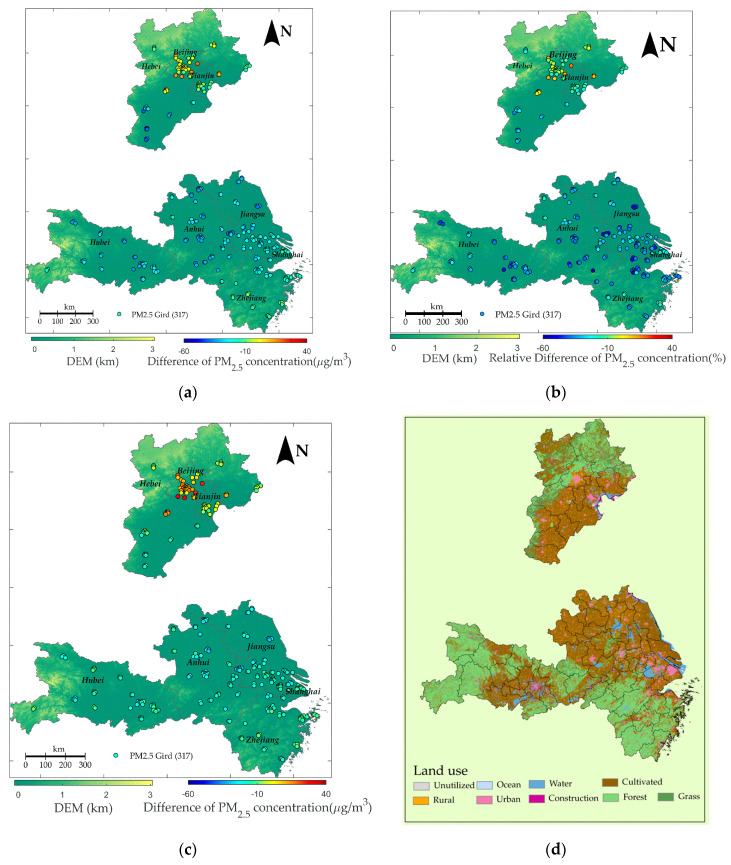
Spatial distribution of (**a**) the mean difference of PM_2.5_ under SD and NSF ($PM_{sd}$ − ${\hat{PM}}_{sd|nsf}$); (**b**) the mean relative difference of PM_2.5_ under SD and NSF (($PM_{sd}$ − ${\hat{PM}}_{sd|nsf}$)/$\;{\hat{PM}}_{sd|nsf}$ × 100); (**c**) the mean difference of PM_2.5_ under SD and NC ($PM_{sd}-{\hat{PM}}_{sd|nc}$); and (**d**) land uses of Beijing–Tianjin–Hebei (BTH), Yangtze River Delta (YRD), and Hubei. The bluer the sites are, the smaller the difference is.

Figure 4a–c shows the spatial distribution of the mean difference and mean relative difference between $PM_{sd}$, ${\hat{PM}}_{sd|nc}$, and ${\hat{PM}}_{sd|nsf}$. The bluer the sites are, the smaller the difference is and the larger the effect of shutting down factories and vehicles during SD on declining PM_2.5_ concentration. Figure 4d shows the land use data in the three regions in 2018, which were collected from the Chinese Resource and Environment Data Cloud Platform (www.resdc.cn/data.aspx?DATAID=264). Through the comparison of Figure 1 and Figure 4, we further explored the spatial effect of fireworks, factories, and vehicles on air pollution.

From the DEM situation shown in Figure 4a and the land uses distribution in Figure 4d, it can be found that South BTH, North YRD, and middle Hubei are low topographically and mainly occupied with cultivated, urban, and rural land and other constructions. Comparatively, other areas of BTH, YRD, and Hubei are high topographically, occupied with grass and forest. Figure 4a shows the spatial distribution of $PM_{sd}$ − ${\hat{PM}}_{sd|nsf}$, which implies the decline degree of the PM_2.5_ concentration induced by the closure of the factories and vehicles compared with the NSF period. The results show that $PM_{sd}$ − ${\hat{\;PM}}_{sd|nsf}$ were generally negative excluding North BTH and tended to be smaller in the cultivated, urban, and rural land. This illustrates that the closure of factories and vehicles generally improved the air quality in south BTH, YRD, and Hubei and the absolute improvement was higher in the areas where human activities were more intense.

Considering that the air pollution was spatially varying, which is indicated by the spatial distribution of mean PM_2.5_ concentration in Figure 1, we also plotted Figure 4b to represent the spatial distribution of the relative effect of the closure of factories and vehicles, which was calculated by ($PM_{sd}$ − ${\hat{PM}}_{sd|nsf}$)/$\;{\hat{PM}}_{sd|nsf}$ × 100. It could be concluded that in South BTH, middle Hubei, and North Central YRD, the relative difference was much smaller, indicating that the relative improvement was also higher in these areas.

Figure 4c shows the spatial distribution of $PM_{sd}-{\hat{PM}}_{sd|nc}$, which implies the difference of PM_2.5_ concentration caused by the difference of the emission scenario during the SD and NC period. Comparing Figure 4a with Figure 4c, we can find that the color of the sites is generally redder in Figure 4c than Figure 4a, especially in South BTH and North YRD. This indicates that in BTH, YRD, and Hubei, $PM_{sd}-{\hat{PM}}_{sd|nc}$ was larger than $PM_{sd}-{\hat{PM}}_{sd|nsf}$, thus ${\hat{PM}}_{sd|nc}$ was smaller than ${\hat{PM}}_{sd|nsf}$, and the difference was more significant in the cultivated, urban, and rural land. This was due to the fact that during NSF, there were many fireworks set off, which may cause much more air pollutants and further aggravate air pollution.

## 4. Discussion

In the context of the isolation of most Chinese cities caused by COVID-19, this paper adopted STRF models to simulate the PM_2.5_ concentration levels of BTH, YRD, and Hubei under the same meteorological conditions but different emission scenarios. This paper further studied the spatiotemporal effect of restricting factories and vehicles during SD and fireworks on air pollution. Three periods were chosen as the study period, namely, the COVID-19 isolation period (21 January 2020–20 February 2020), the previous month before the isolation period (21 December 2019–20 December 2020), and the same period in the lunar calendar of last year (1 February 2019–3 March 2019), to represent the emission scenarios of SD, NC, and NSF, respectively. Based on the meteorological data and PM_2.5_ observations of the NC and NSF periods, hourly STRF models (${\mathrm{Model}}_{\mathrm{nc}}$ and ${\mathrm{Model}}_{\mathrm{nsf}}$) were developed to simulate the correlation between PM_2.5_ and meteorological variables under the specific emission scenarios of NC and NSF. Then, the meteorological data of the SD period was inputted into ${\mathrm{Model}}_{\mathrm{nc}}$ and ${\mathrm{Model}}_{\mathrm{nsf}}$, respectively. We obtained the simulated PM_2.5_ concentration level under the meteorological condition of SD and emission scenarios of NC (${\hat{PM}}_{sd|nc}$) and NSF (${\hat{PM}}_{sd|nsf}$). The results presented in Table 3 prove the reliability of the developed STRF models. By comparing $PM_{sd}$ (PM_2.5_ concentration observations under the meteorological and emission condition of the SD period) with ${\hat{PM}}_{sd|nc}$ and ${\hat{PM}}_{sd|nsf}$, we explored the spatiotemporal effect of shutting down factories and vehicles and fireworks on the PM_2.5_ pollution level, providing new and detailed support for the implementation of emission control policies.

According to the results, the effect of shutting down factories and vehicles has obvious spatiotemporal patterns. (a) From the perspective of the temporal analysis, Table 4 shows that compared with the NSF period, the emission control measures during the SD period reduced the concentrations of PM_2.5_ by 18.57, 16.22, 25.00, and 19.07 $\mu g/m^{3}$ at 02:00, 08:00, 14:00, and 20:00, respectively. The impact was largest at 14:00 and 20:00, followed by 02:00 and 08:00. The decrease in 08:00 was mainly due to the reduction of traffic flow during morning rush-hour, which has been confirmed to be an important contributor of the pollution at this time [10]. However, the restriction of factories and vehicles caused a larger decrease of PM_2.5_ concentration at 14:00 and 20:00. This is owed much to the great reduction of human activities during the daytime. Zhou et al. found that human activities are complicated, including all kinds of industry, shopping, restaurant, entertainment, etc. These have led to industrial agglomeration and further generated various exhaust emissions and even cumulative emission, resulting in a greater impact on the concentration of pollution in the afternoon and at night [49]. Thus, during the SD period when human daytime outside activities were reduced, the PM_2.5_ concentration of 14:00 and 20:00 declined significantly, especially for 14:00 when normally there are many human activities. Wang et al. also found that the factor of vehicular/industrial emissions contributed more in nighttime than in daytime and there was a peak in morning rush-hour. These are consistent with the results of this study. However, their results showed that the contribution of vehicular/industrial emissions was the least at around 14:00, which is contrary to the conclusion of this paper. This may be due to the fact that the data used in the study of Wang et al. were collected from the single urban monitoring station surrounded by commercial properties and residential dwellings, while this study considered more stations under various backgrounds, such as industrial and rural environments. Nevertheless, the obvious diurnal variation trend observed both in this paper and the study of Wang et al. illustrates that the influential factors of air pollution change with hours and the impact degree of each socioeconomic factor on air pollution is also varying. Thus, hourly analysis of the major components and sources are necessary to further alleviate air pollution. (b) From the perspective of spatial analysis, previous studies have shown that elevation and land use are vital influencing factors of PM_2.5_ [50,51]. In this paper, Table 4 and Figure 4 show a high correlation between the terrain characteristics, land uses, PM_2.5_ pollution levels, and the spatial effects of shutting down factories and vehicles. In general, the low-lying areas (including Southeast BTH, Northern YRD, and Middle Hubei) are occupied with cultivated, urban, and rural land. In these areas, the air pollution was more severe and $PM_{sd}$ − ${\hat{PM}}_{sd|nsf}$ as well as (($PM_{sd}-{\hat{PM}}_{sd|nsf}$)/$\;{\hat{PM}}_{sd|nsf}$ × 100) were smaller. The severe air pollution in these areas was highly correlated with rapid economic development. Therefore, restricting factories and vehicles during SD effectively reduced the emission of air pollutants and thus had a great effect on the alleviation of air pollution. In contrast, the high-lying areas (including Northwest BTH, Southern YRD, and the west and east sides of Hubei) have grasslands and forest, inducing better air quality and larger $PM_{sd}-{\hat{PM}}_{sd|nsf}$ and (($PM_{sd}-{\hat{PM}}_{sd|nsf}$)/$\;{\hat{PM}}_{sd|nsf}\;$× 100), indicating that shutting down factories and vehicles during SD did not greatly affect these areas. This is consistent with the conclusions in the studies of Wang et al. and Lin et al., which illustrated that the increasing population and rapid urban expansion had an adverse impact on air quality [50,51].

The spatial and temporal effects of shutting down factories and vehicles are complex and interweaving. First, the $PM_{sd}-{\hat{PM}}_{sd|nsf}$ results in Table 4 show that the restriction of factories and vehicles during SD led to a decline of PM_2.5_ concentration by 8.20, 23.52, and 27.05$\;\mu g/m^{3}$ for BTH, YRD, and Hubei, respectively. All hourly results of $PM_{sd}-{\hat{PM}}_{sd|nsf}$ in YRD and Hubei exceeded the MAE range, indicating that shutting down factories and vehicles during SD led to a significant decline in PM_2.5_ concentration. The results indicate that the restriction of factories and vehicles during SD was generally effective in declining the PM_2.5_ in BTH, YRD, and Hubei. However, the effect showed significant spatial heterogeneity across different regions. The PM_2.5_ concentrations of Hubei declined more than that of YRD. This is in agreement with the trend illustrated in the study of Xu et al. that the impacts of private cars on PM_2.5_ emissions decrease continuously from east to west [41]. However, the air pollution in BTH was more severe and $PM_{sd}-{\hat{PM}}_{sd|nsf}$ was the largest. This may be explained by other important emission sources and unfavorable weather conditions. (1) Coal combustion is an important emission source of PM_2.5_ in BTH. Andersson et al. and Zhou et al. have proved that the contribution of vehicle emissions was lower in BTH than southern cities; in contrast, coal combustion occupied a higher proportion of PM_2.5_ in BTH [52,53]. This is not surprising as coal combustion is commonly used for residential heating in BTH during winter, while there is no central heating for southern cities such as YRD and Hubei. Zhang et al. also proved that the residential sector is a notable contributor to PM_2.5_ in northern China during heating season [25]. The residuals of coal combustion contribute a lot to the formation of PM_2.5_ of BTH, meaning the effect of shutting down factories and vehicles was smaller in BTH than YRD and Hubei. (2) The synoptic patterns and meteorological elements are also vital inducements of the formation of severe haze in BTH. Compared with Hubei and YRD, with relatively flat terrain, BTH is high in the northwest and low in the southeast, inducing evident mountain-valley breeze circulation. Bei et al. have studied the impact of local circulation on air pollution [54,55,56]. During the daytime, the valley breeze (southerly or easterly wind) increases and the pollutants are transported from urban to mountain areas. At night, Beijing is affected by westerly wind down from Taihang Mountains and northerly wind down from Yanshan Mountains (mountain breeze), and pollutants are delivered back to urban areas. The mountain–valley breeze circulation facilitates the accumulation of pollutants, meaning the pollution level of BTH is affected by the total pollutant emissions that have accumulated over a long period of time, so shutting down factories and vehicles did not bring a significant decrease for the PM_2.5_ of BTH.

Second, according to the studies of Zhang et al. and Ding et al., which compared the air pollution between 2013 and 2017 in a coarse temporal resolution, the emission control policies significantly contributed to the declining of PM_2.5_ concentrations and PM_2.5_-mortality during this period [25,26]. This paper proved the effect of the emission control measures and further supplemented more detailed analysis of the daily variations of the effect. We found that there was a delay for the restriction of factories and vehicles in declining PM_2.5_ concentrations. Figure 3 shows that the delay in shutting down factories and vehicles had a significant effect on the improvement of air quality during SD, and the delay time varied among different regions. For YRD, it took 23 days to observe the measured PM_2.5_ concentration ($PM_{sd}$) as significantly less than ${\hat{PM}}_{sd|nsf}$ and ${\hat{PM}}_{sd|nc}$; for Hubei, it took 17 days. Many studies have confirmed that a large part of PM_2.5_ is formed by the anthropogenic emissions, and the meteorological condition, especially wind, plays an important role in the transport and dispersion of the air pollutant [57]. Considering that the discharge of pollutants greatly exceeds the environmental capacity, the daily pollutants cannot be purified in time. Therefore, PM_2.5_ concentration is not only affected by the air pollutant emission amount of the current day (denoted as E(t)), but is also correlated with the cumulative emission amount during a previous period (denoted as $\sum_{i=0}^{d}E\left(t-i\right)$). After closing the factories and vehicles, the daily emissions E(t) were reduced a lot, however influenced by the air pollutants discharged in the preceding period and emissions from other sources, the cumulative emissions $\sum_{i=0}^{d}E\left(t-i\right)$ were not reduced much in ratio. Thus, PM_2.5_ concentration did not show an immediate great decrease. Obviously, the decline ratio of $\sum_{i=0}^{d}E\left(t-i\right)$ is negatively correlated with d and as the number of days that factories and vehicles are closed increases, $\sum_{i=0}^{d}E\left(t-i\right)$ decreases much more and the impact of closing factories and vehicles gradually increases. Hence, the phenomenon that there was a delay time in PM_2.5_ to show a great decrease is shown in Figure 3.

**Figure 5:**
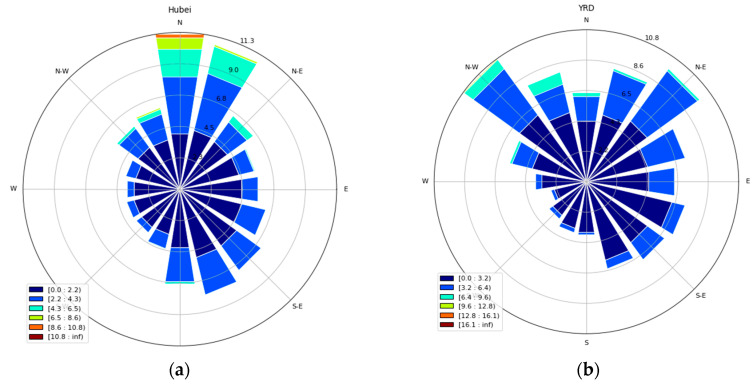
Distribution of wind speed and wind direction of (**a**) Hubei and (**b**) YRD during the SD period.

The reason why the delay time is different in Hubei and YRD can be analyzed from three aspects. (1) The pollution situation is complex and different for Hubei and YRD. For one month before the outbreak of COVID-19, that is, the NC period, the average PM_2.5_ concentration of Hubei was 75.83 $\mu g/m^{3}$, which was higher than that of YRD (60.57 $\mu g/m^{3}$). This implies that the emission of pollutants in Hubei may be higher than that in YRD. According to the relative decline proportion shown in Figure 4b, the average decline proportion of PM_2.5_ concentration in Hubei and the YRD region was 31.88% and 25.31%, respectively. This implies that the air pollution in Hubei was more serious than that in YRD and the contribution of vehicle and factory emissions to local pollution was higher. Therefore, the influence of the closure of factories and vehicles during SD was higher in Hubei and the delay time was shorter. (2) From the view of the topographic and climatic conditions, the natural conditions are different for Hubei and YRD region. A previous study has shown that wind is a key influencing factor of PM_2.5_ as low wind speeds suppressed the removal of PM_2.5_ and high frequency of wind of a proper direction can lead to low PM_2.5_ concentration [25]. Figure 5 shows the distribution of wind speed and wind direction of Hubei and YRD during the SD period. The dominant wind direction in Hubei was the north wind, while the dominant wind direction in YRD was the northeast and northwest wind. It is worth noting that Hubei province is larger in the east–west span than the north–south span and there is no significant change of DEM in the north–south direction. However, the YRD region is longer in the north–south direction than the east–west direction and DEM shows a higher tendency from north to south. Under the dominant northwest and northeast wind, such terrain conditions of YRD mean that the timely spread outside of the air pollutant is difficult. Therefore, the PM_2.5_ concentration of YRD is influenced by the cumulative emissions for a longer time (i.e., d is longer for YRD), hence $\sum_{i=0}^{d}E\left(t-i\right)$ decreased more slowly. This is one of the reasons why the delay time of the YRD region was longer than that in the Hubei region. (3) The restriction degree of factories and vehicles was different. Hubei is the worst hit region by COVID-19 and the number and proportion of infectious cases in Hubei province, especially in Wuhan city, are significantly higher than those in other regions. According to the reports from provincial and municipal health commissions, by the end of 19:00, 7 February 2020, the cumulative number of infections in Hubei was 22,112, while the highest in the rest of the country was in Guangdong, with 1034 cases having been reported. The severe epidemic situation induced the Hubei government to adopt the strictest policies to restrict people going out and factories opening. Under this background, the public’s sense of spontaneous home isolation is even stronger. All of these objective measures and subjective consciousness made human activities in Hubei decline to the greatest extent and the emission of air pollutants declined much more. Thus, the time it took for shutting down factories and vehicles to significantly affect the amount of air pollutants was shorter.

The fireworks aggravated air pollution and the impact showed spatiotemporal heterogeneity. By collecting the PM_2.5_ concentration samples from specific sites before and after the Spring Festival and analyzing their chemical characteristics, Wang et al. and Feng et al. found that during the Spring Festival, the concentrations of K^+^, Mg^2+^, and Al resulting from firework displays increased significantly [16,17]. However, limited by the spatial range of few monitoring stations, these studies could not analyze the spatial heterogeneity of the effect of fireworks. This paper analyzed the effect of fireworks on a larger spatial scale. According to the comparison of $PM_{sd}-{\hat{PM}}_{sd|nc}$ and $PM_{sd}-{\hat{PM}}_{sd|nsf}$ in Table 4, the fireworks improved the PM_2.5_ concentration by 10.94, 6.03, and 11.46 $\mu g/m^{3}$ on average for BTH, YRD, and Hubei, respectively. The spatial distribution of $PM_{sd}$ − ${\hat{PM}}_{sd|nc}$ and $PM_{sd}$ − ${\hat{PM}}_{sd|nsf}$ in Figure 4c shows that the impact of fireworks was more significant in the cultivated, urban, and rural land. We can infer that the strict implementation of a policy prohibiting fireworks is still necessary as it reduces the PM_2.5_ concentration in the cultivated, urban, and rural areas.

China is currently undergoing a strategic transition from economic development to environmental development and a series of policies and measures for air pollutant emission control have been implemented [58,59,60]. For example, the government has invested heavily in the production of alternative energy, including solar, wind, hydro, and nuclear production [40]. Moreover, to reduce the emission of vehicles, the government launched strict national standards for the discharge of pollutants and implemented traffic control measures during rush hours for working days [61,62]. In terms of industrial areas, factories with heavy emissions were shut down and were required to renovate their emission methods [63]. These policies highlight the determination of the Chinese government to improve air quality. Some studies have confirmed the effectiveness of these air pollutant control measures [25,26,35,36,64] and this paper further explored the spatiotemporal effect of shutting down factories and vehicles during SD and the effect of fireworks on air pollution using spatial and temporal scales. The analysis results provided evidence that shutting down factories and vehicles and reducing fireworks can effectively alleviate air pollution, especially in the cultivation, urban, and rural lands. The results of studies on spatiotemporal heterogeneity can offer more guidance for the government to implement policies in different cities.

## 5. Conclusions

The isolation of most Chinese cities caused by COVID-19 provided the condition of studying the spatiotemporal effect of shutting down factories and vehicles as well as fireworks on air pollution. Based on the PM_2.5_ concentration and meteorological data during NC (21 December 2019–20 January 2020) and NSF (1 February 2019–3 March 2019) of BTH, YRD, and Hubei, a series of hourly STRF models were developed to simulate the correlation between PM_2.5_ with meteorological condition under a specific emission scenario and specific hour. Then, the meteorological data during SD (21 January 2020–20 February 2020) were inputted into the STRF models to predict the expected PM_2.5_ pollution level under NC and NSF. The reliability of the STRF models was verified through 10-fold cross validation experiments. By comparing the observed PM_2.5_ concentration during SD with the predicted PM_2.5_ concentration under emission scenarios of NC and NSF, this paper explored the impact of shutting down factories and vehicles during SD and fireworks on PM_2.5_ concentration from multiple spatial and temporal perspectives. The main conclusions are as follows:

(1) The impacts of restricting factories and vehicles on declining PM_2.5_ concentration shows obvious diurnal variations. Due to the reduction of traffic flow and human activities during the daytime, the cumulative emissions were reduced significantly. Consequently, the PM_2.5_ concentration at 14:00 was reduced the most (25.00 $\mu g/m^{3}$), followed by 20:00 and 02:00 during the nighttime (19.07 and 18.57 $\mu g/m^{3}$, respectively), and the air pollution at 08:00 during the morning rush-hour was also alleviated to some extent (16.22 $\mu g/m^{3}$).
(2) The air quality is not only affected by the emissions of current day, but is also influenced by the cumulative emissions discharged in the previous period. Therefore, there was a delay in the time it took for the restriction of factories and vehicles to have a significant effect on improving air quality and the delay time for Hubei and YRD were 17 and 23 days, respectively.
(3) The effect of restricting factories and vehicles shows obvious regional differences. Due to the discrepancies in the composition of PM_2.5_, the contribution ratio of industrial and vehicular emissions, and the geographic conditions, BTH, YRD, and Hubei experienced a 8.20, 23.52, and 27.05$\;\mu g/m^{3}$ decrease of PM_2.5_ concentration, respectively. On average, the air quality of Hubei was improved the most significantly and the fastest, followed by YRD. The air quality of BTH was improved the slightest because of the emissions from coal combustion and unfavorable meteorological conditions for air pollutants to be spread.
(4) On account of the impact of more intensive human activities, cultivated, urban, and rural land are more sensitive to the emissions from factories, vehicles, and fireworks. The air quality in these areas was improved much more significantly than the forest and grass land after restricting the above emission sources.

These fine-grained spatiotemporal results have important research and policy implications. First, more attention should be paid to the hourly heterogeneity analysis of the effect of various emission control measures. The restriction of factories and vehicles had the most significant effect on improving air quality at 14:00 compared with three other hours. Similarly, the effect of the restriction of other emission sources, such as cooking and coal combustion, may also have hourly heterogeneity. In order to implement more targeted and effective policies aiming at reducing public exposure, especially during the human activity rush-hour, more hourly analysis is needed to further investigate the influential factors of air pollution and the hourly heterogeneity.

Second, the government should combine long-term and short-term policies together in practice and consider more about the daily variations of the effect of various emission-reduction measures. The strict restriction of factories and vehicles did not alleviate the air pollution immediately; instead, the air quality did not show significant improvement until several days later. Therefore, when there is a heavy haze, the government cannot expect the temporary restriction of industries and cars to make a great difference on the air quality in a short time. In contrast, the restriction of factories and vehicles must be insisted on for a long time to obtain a significant effect of improving air quality, including developing technologies of cleaning up exhaust gas from vehicles and factories and promoting the emission standards. Moreover, when there are adverse meteorological conditions, such as steady wind and temperature inversion, and heavy air pollution is induced, some short-acting measures need to be combined, such as using artificial precipitation to accelerate the sedimentation of pollutant. In order to better combine the long-term and short-term policies, more investigation of the variation tendency of the effect of various measures are needed.

Finally, the policies aiming at reducing PM_2.5_ emissions should be different in the three regions. The restriction of factories and vehicles had different impacts on air pollution across three regions, so the discrepancies should be considered in implementing relevant policies. For Hubei and YRD, there is need to control the excessive increase of ownerships of private vehicles, promote public transport, and support the usage of new energy for cars while alternative clean energy and technologies of coal combustion need to be greatly improved in BTH.

The more fine-grained analysis results of this paper could provide more evidence that shutting down factories and vehicles as well as the control of fireworks play important roles in the improvement of air quality. The conclusions are valuable for exploring the spatiotemporal patterns of their effect on air pollution and public exposure, especially for investigating the hourly heterogeneity of the impact and the delay time of bringing significant change regarding air pollution. Along with the spatiotemporal patterns of human activities, the conclusions provide help in the implementation of emission control policies and measures to protect public health. However, this paper had some limitations. First, studies on the retrieval methods of air pollutants concentration are many [46,65,66] and thus, more accurate PM_2.5_ concentration retrieval models can be investigated further to improve the simulation accuracy, such as the widely used neural networks. Second, due to the difficulties and privacy issues in collecting relevant information, it is hard to obtain concrete and accurate emission inventories during a specific period. Therefore, the conclusion can provide more support on how factories and vehicles affect air pollution if the concrete inventory of factories and vehicles was collected. This is also the future research direction of our future studies.

## Figures and Tables

**Table 1:** Emission scenarios of the study periods.

No.	Time	Emission Scenarios	Abbreviation	Fireworks	Heating	Factories and Vehicles
1	21 January 2020–20 February 2020	Shut Down factories and vehicles	SD	Yes	Normal	Shut Down
2	21 December 2019–20 January 2020	Normal Commute	NC	None	Normal	Normal
3	1 February 2019–3 March 2020	Normal Spring Festival	NSF	Yes	Normal	Normal

**Table 2:** Meteorological and emission background of $PM_{sd}$, ${\hat{PM}}_{sd|nsf}$, and ${\hat{PM}}_{sd|nc}$.

Data	Type	Meteorological Scenario	Emission Scenario
$PM_{sd}$	Observed	SD	SD
${\hat{PM}}_{sd|nsf}$	Predicted	SD	NSF
${\hat{PM}}_{sd|nc}$	Predicted	SD	NC

**Table 3:** The average prediction accuracy of 10-fold cross validation experiments of STRF models in different regions.

Hour (BJT)	Region	Normal Spring Festival Period				Normal Commute Period			
		Obs.	RMSE	MAE	R^2^	Obs.	RMSE	MAE	R^2^
02:00	BTH	96.04	35.06	20.15	0.83	78.88	25.69	16.72	0.85
	YRD	59.69	16.15	10.54	0.87	57.73	15.54	10.42	0.88
	Hubei	70.78	19.48	12.39	0.79	65.18	15.61	11.40	0.82
	All	71.47	23.88	13.49	0.84	64.73	19.01	12.32	0.87
08:00	BTH	84.22	27.27	17.13	0.85	71.82	25.56	16.22	0.84
	YRD	59.02	18.69	11.22	0.82	57.51	15.47	10.58	0.87
	Hubei	70.40	20.37	13.05	0.80	59.82	15.34	11.44	0.79
	All	67.71	21.73	13.15	0.84	61.86	18.88	12.28	0.85
14:00	BTH	70.22	21.49	13.25	0.89	66.69	20.89	13.36	0.89
	YRD	57.12	14.25	9.44	0.88	55.59	13.25	9.24	0.89
	Hubei	67.48	16.22	11.41	0.82	61.49	14.15	10.43	0.83
	All	62.27	16.93	10.81	0.88	59.54	15.92	10.56	0.89
20:00	BTH	85.96	25.53	15.47	0.86	79.87	24.06	16.13	0.85
	YRD	60.96	17.30	11.57	0.84	59.92	15.16	10.45	0.87
	Hubei	67.01	16.79	11.83	0.80	68.25	14.66	10.99	0.84
	All	68.84	19.96	12.70	0.85	66.71	18.10	12.13	0.86

BTH: Beijing–Tianjin–Hebei; YRD: Yangtze River Delta; RMSE: root mean square error; MAE: mean absolute error; STRF: spatiotemporal random forest.

**Table 4:** Average difference of the PM_2.5_ concentration under different emission scenarios.

Area	$PM_{sd}$ − ${\hat{PM}}_{sd|nsf}$					$PM_{sd}$ − ${\hat{PM}}_{sd|nc}$				
	02:00	08:00	14:00	20:00	All	02:00	08:00	14:00	20:00	All
BTH	−11.11	−11.54	−4.67	−5.47	−8.20	4.01	4.09	1.61	1.25	2.74
YRD	−20.93	−16.75	−32.12	−24.26	−23.52	−17.32	−13.21	−19.12	−20.30	−17.49
Hubei	−23.75	−23.33	−36.28	−24.83	−27.05	−11.98	−9.31	−18.08	−23.00	−15.59
All	−18.57	−16.22	−25.00	−19.07		−10.57	−7.80	−13.15	−14.63	
